# Are Histrionic Personality Traits Associated with Irritability during Conscious Sedation Endoscopy?

**DOI:** 10.1155/2015/702492

**Published:** 2015-04-12

**Authors:** Sang Shin Lee, Hyung Hun Kim, Hyo Jung Park

**Affiliations:** ^1^Department of Psychiatry, College of Medicine, Kosin University, Busan 602-702, Republic of Korea; ^2^Department of Internal Medicine, College of Medicine, The Catholic University of Korea, Seoul 137-701, Republic of Korea

## Abstract

*Aim*. We aimed to evaluate whether histrionic personality traits are associated with irritability during conscious sedation endoscopy (CSE). *Materials and Methods*. A prospective cross-sectional study was planned. Irritability during CSE was classified into five grades: 0, no response; I, minimal movement; II, moderate movement; III, severe movement; IV, fighting against procedure. Patients in grades III and IV were defined as the irritable group. Participants were required to complete questionnaire sheet assessing the extent of histrionic personality traits, extraversion-introversion, and current psychological status. The present authors also collected basic sociodemographic data including alcohol use history. *Results*. A total of 32 irritable patients and 32 stable patients were analyzed. The histrionic personality trait score of the irritable group was higher than that of the stable group (9.5 ± 3.1 versus 6.9 ± 2.9; *P* = 0.001), as was the anxiety score (52.8 ± 8.6 versus 46.1 ± 9.6; *P* = 0.004). Heavy alcohol use was more frequently observed in the irritable group (65.6% versus 28.1%; *P* = 0.003). In multivariate analysis, all these three factors were independently correlated with irritability during CSE. *Conclusion*. This study revealed that histrionic personality traits, anxiety, and heavy alcohol use can affect irritability during CSE.

## 1. Introduction

Sedation endoscopy employs sedative premedication to induce conscious sedation for a comfortable endoscopy [1–3]. Sedation has repeatedly been shown to contribute to superior patient satisfaction, comfort, and willingness to undergo repeat procedures [4]. Many patients prefer conscious sedation endoscopy (CSE) to ensure their safety, comfort, and cooperation. Midazolam is the sedative most widely used for this procedure because it has many advantages, such as a short half-life, faster onset of sedation, and an excellent sedative/hypnotic effect without any particular side effects such as vasculitis. A low dose of midazolam is recommended as premedication for endoscopy because of a potential risk of hypotension and hypoxia in a high dose of midazolam [5–7].

Although midazolam is a very effective premedication for CSE, an unexpected irritable response to endoscopy after adequate sedation can make the practice difficult and can even endanger the patient. Irritable responses cover a wide range from slight resistance to violent behavior such as pulling out the endoscope with their own hands. One of the dangerous aspects of irritable response to CSE is that patients usually cannot control their action. Endoscopists experience this situation often and have become familiar with behavioral management like physical restraints for irritable and violent patients during CSE.

An irritable response could be mistaken for a paradoxical response to midazolam, but it differs from a paradoxical response in several ways. Paradoxical responses to midazolam include symptoms of agitation, restlessness, uncontrollable shaking, and stiffening and jerking of the arms and legs unexpectedly [8]. This paradoxical excitement occurs in less than 1% of all patients who receive midazolam, and it can be reversed with flumazenil [9, 10]. In contrast, irritability during CSE refers to an irritable behavioral response to endoscopic stimuli after adequate sedation, which seems not to be uncommon; however, there are currently no epidemiologic data available on this topic.

Based on our clinical experiences, people who have shown a more irritable response during CSE appear to be nervous, emotionally labile, inconsistent in their somatic symptoms, and intolerant of relatively minor pain in an alert state. Such people have been described as having “hysteria” or a “hysterical personality” [11]. However, this concept of “hysteria” or a “hysterical personality” grounded on the psychoanalytic theory is no longer valid in the modern psychiatric disorder classification system of the Diagnostic and Statistical Manual of Mental Disorders (DSM) [12], which should be required for the purpose of diagnosis especially in regard to clinical research. This traditional concept of “hysteria” or “hysterical personality” is reflected in the somatoform disorders and cluster B personality disorders such as the histrionic, borderline, antisocial, and narcissistic personality disorders in the DSM-IV-TR [11–13].

We hypothesized that irritable response to CSE might be related to histrionic personality traits of cluster B personalities based on the DSM-IV-TR classification. To verify this hypothesis, we examined the psychological background (e.g., the extent of histrionic personality traits and current psychological state) and physical background of patients who underwent midazolam-induced CSE using a prospective design.

## 2. Materials and Methods

### 2.1. Study Design

Participants who were scheduled to undergo midazolam-induced CSE at the Gastrointestinal Center of Kosin University Gospel Hospital, Busan, South Korea, were invited to participate in this study. Written informed consent was obtained from all participants. The exclusion criteria included inability to understand written informed consent, severe cognitive impairment, severe cardiopulmonary disease, pregnancy, allergy to midazolam, medication affecting midazolam metabolism, and emergent endoscopy. This prospective observational study was approved by the institutional review board of Kosin University College of Medicine (12-014) and registered with the Clinical Research Information Service (KCT0000621).

### 2.2. Sociodemographic and Clinical Data

In addition to basic sociodemographic data, clinical data including history of alcohol use and cigarette smoking, the reason for endoscopic examination, and comorbid physical diseases were also collected. We defined heavy drinkers as participants who reported drinking at least 3 bottles of Korean distilled liquor (soju) a week.

### 2.3. Psychological Assessment

In order to assess personality which is defined as the life-long stable pattern of an individual's inner experience, we modified the histrionic personality section in the Structured Clinical Interview for DSM-IV-TR Axis II Personality Disorder (SCID-II) Personality Questionnaire [14]. Additionally, the Extraversion-Introversion scale of the Myers-Briggs Type Indicator (MBTI) was applied [15]. SCID-II and MBTI represent an enduring personality trait of participants. On the contrary, the Symptom Checklist-90-Revised (SCL-90-R) [16] reflects a current psychological state in that period of the test. All questionnaires were completed before the CSE. The entire psychological scoring process was conducted by the psychologist (H. J. Park) among the authors. H. J. Park was blinded to the grades of irritability during CSE.

#### 2.3.1. Modified Form of the SCID-II Personality Questionnaire

The psychiatrist (S. S. Lee) and psychologist (H. J. Park) of the authors modified the SCID-II Personality Questionnaire to assess the extent of histrionic personality traits. The SCID-II is a semistructured clinical interview instrument for the diagnosis of personality disorders based on the DSM-IV. Although investigators and clinicians can use all sections of the SCID-II, they can also choose to use only selected sections relevant to the specific purpose of their research to examine specific personality traits [14]. SCID-II Personality Questionnaire is a self-report scale for his/her own personality and is useful as screening tool for DSM-IV-TR personality disorders [17]. We selected and used the section of histrionic personality (items 66–72) from the SCID-II Personality Questionnaire. The original version of SCID-II Personality Questionnaire requires a dichotomous yes/no response in order to categorical diagnosis of personality disorder. Because we aimed to estimate the influence of the extent of histrionic personality on the irritability during CSE, not to diagnose a histrionic personality, we needed to modify a yes/no format of the original version to a Likert-type one: 1, always wrong; 2, usually wrong; 3, average; 4, usually true; 5, always true. For example, a participant was required to answer item 66, “Do you like to be the center of attention?” with the number of Likert-type scale which was the most relevant to his/her own attitude. We used the Korean version of the SCID-II Personality Questionnaire [18]. Higher scores indicate a greater tendency toward histrionic personality traits.

#### 2.3.2. Extraversion-Introversion of the MBTI

The MBTI is a personality inventory theoretically based on the psychological typology of Carl Gustav Jung's analytic psychology [15]. According to C. G. Jung, “hysteria” is frequently founded in the extraverted person [19]. In line with this, we expected that participants with extraverted would be more irritable during CSE than with introverted. We used the extraversion and introversion section of the MBTI to distinguish the general attitude type of the participants. The scale consists of 21 items, and the subjects were asked to select one of two statements that describe themselves more appropriately [20]. The respondents were classified into two groups of the extravert and the introvert.

#### 2.3.3. Symptom Checklist-90-Revised

The SCL-90-R is a self-reported scale to assess an individual's current psychological state spanning the week prior to the assessment [16]. The SCL-90-R consists of 90 items that are categorized into 9 symptom dimensions: somatization, obsession-compulsion, interpersonal sensitivity, depression, anxiety, hostility, phobic anxiety, paranoid ideation, and psychoticism. The participants were required to complete the Korean version of the SCL-90-R [21]. We estimated standardized *T*-scores for each symptom dimension. Higher scores on the SCL-90-R indicate a higher tendency for the relevant symptom dimension.

### 2.4. Procedure

Prior to CSE, the participants were administered with midazolam intravenously under close monitoring of vital signs. A loading midazolam dose of 0.05 mg/kg was injected over 1-2 minutes and then an additional 1 mg was repeatedly injected over intervals of 2 minutes until a satisfactory conscious sedation state was achieved in terms of Richmond Agitation-Sedation Scale (RASS) scores of −1 to −3 (Table 1) [22].

### 2.5. Measuring Irritability during CSE

We created the Irritability during conscious sedation endoscopy (I-CSE) scale to measure behavioral responses to the advancing endoscopic fiber. The I-CSE scale classifies the irritability of participants into five grades from “no motor response (Grade 0)” to “fighting against procedure (Grade IV)” depending on the extent of upper/lower/trunk movements and the number of assistants needed to control the participant's movements during the endoscopic procedure (Table 2). Participants who showed an irritability of Grade III or Grade IV on the I-CSE were classified into the irritable groups. H. H. Kim, an experienced endoscopist, rated the patient's extent of irritability using the I-CSE.

### 2.6. Statistical Analysis

Statistical analysis was performed using the Statistical Package for the Social Sciences software (SPSS version 16.0, Chicago, IL, USA). We analyzed baseline data such as age, gender, body mass index, basic laboratory findings, purpose of endoscopy, and comorbidities. Cronbach's *α* was calculated for the modified the SCID-II Personality Questionnaire in order to test an internal consistency as reliability. Differences of mean scores for histrionic personality traits and symptom dimensions of the SCL90-R between the two groups were analyzed using independent samples *t*-test. Differences of frequency for extraverts, heavy drinkers, smokers, and previous endoscopic experiences between the two groups were tested using the *χ* ^2^ test. Pearson's correlation test was performed to examine a correlation or collinearity between two continuous variables. Statistical significance was set at a *P* value of <0.05, and if there was more than one factor with a significant difference in univariate analysis, multivariate analysis was performed using a logistic regression model.

## 3. Results

### 3.1. Flow of the Study

The prospective, case-control study included a total of 32 patients in the irritable group and 32 patients in the stable group from December 2012 to February 2013. A total of 109 patients were screened for the study. One patient did not provide consent and 5 were excluded due to a drug interaction. A total of 103 patients completed psychological questionnaires and underwent endoscopic examination. Among these 103 patients, 71 (68.9%) were classified in the stable group and 32 (31.1%) were classified in the irritable group. We randomly selected 32 patients from the stable group by using a table of random numbers for comparison with the 32 participants in the irritable group.

### 3.2. Baseline Characteristics

There were no significant differences between the irritable group and the stable group in terms of age, gender, body mass index, or laboratory findings except ALT level (Table 3). Half (50.0%) of the patients underwent endoscopic examination for screening and 43.7% underwent the procedure as a regular follow-up after an endoscopic submucosal dissection. A total of 9 (14.1%) patients had diabetes mellitus, 17 (26.6%) had hypertension, and 26 (40.6%) had fatty liver disease. There were no differences between the two groups in terms of the purpose of the endoscopic examination or comorbidities. In a basic laboratory test, ALT was higher in the irritable group (25.0 ± 8.4 versus 20.0 ± 6.0, *P* = 0.008).

### 3.3. Dose of Midazolam and Richmond Agitation-Sedation Scale

The dose of midazolam for initial sedation was slightly higher in the irritable group but not statistically different (3.31 ± 0.69 versus 3.17 ± 0.73, *P* = 0.434). RASS grades did not differ between the two groups (Table 4). However, an additional dose of midazolam after advancing the endoscope was significantly higher in the irritable group (1.31 ± 2.51 versus 0.67 ± 1.02, *P* = 0.039) as essentially anticipated.

### 3.4. Modified SCID-II Personality Questionnaire and the Extraversion-Introversion of MBTI

Cronbach's *α* for modified SCID-II Personality Questionnaire was 0.736. This indicated that the modified SCID-II Personality Questionnaire used in the present study had an internal consistency as a psychometric measure [23]. Histrionic trait scores were significantly higher for the irritable group than for the stable group (9.5 ± 3.1 versus 6.9 ± 2.9, *P* = 0.001, Table 5). More patients in the irritable group were found to be extraverts, but this difference was not significant (53.1% versus 37.5%, *P* = 0.209).

### 3.5. Symptom Checklist-90-Revised

The irritable group reported a higher anxiety score than the stable group (52.8 ± 8.6 versus 46.1 ± 9.6, *P* = 0.004). There was no significant difference in score between the irritable and stable groups for other symptom domains (Table 5). Moreover we conducted Pearson's correlation analysis to investigate collinearity between histrionic personality trait and anxiety. There were no statistically significant collinearity between the two variables for total pooled sample (*r* = 0.124, *P* = 0.330, VIF = 1.0).

### 3.6. Social Habits, Previous Endoscopy Experience

Heavy alcohol users were more common in the irritable group (65.6% versus 28.1%, *P* = 0.003, Table 3). Smoking was less frequently observed in the irritable group, but there was no statistical difference (18.8% [irritable] versus 40.6% [stable], *P* = 0.055). The irritable group had a greater number of previous endoscopic examination experiences, but this difference was not statistically significant (93.8% versus 84.4%, *P* = 0.230).

### 3.7. Multivariate Analysis

Pearson's correlation analysis showed that histrionic personality trait and anxiety had a significant positive correlation with I-CSE for the total pooled sample (*r* = 0.334  *P* < 0.01; *r* = 0.262  *P* < 0.05, resp., Table 6). Combined with basic demographic variables of age and gender, histrionic personality trait, anxiety, and heavy alcohol use that showed statistically significant differences in the univariate analysis were entered in the logistic regression analysis with forward selection. Although a variable of ALT was also significantly different between two groups, it is not considered to be relevant with the aims of this study and could worsen the statistical probability. Hence, the variable of ALT was not included as the covariate in a multivariate logistic regression.

Logistic analysis showed that a histrionic personality was a statistically significant associative factor for irritability during endoscopic examination (odds ratio = 1.294, *P* = 0.015, 95% CI 1.052–1.590) as well as anxiety and heavy alcohol use. Heavy alcohol use was the most powerful variable for I-CSE (Table 7).

## 4. Discussion

Midazolam can induce sedation, more specifically, depression or lowering of the level of consciousness which is essential in CSE. Irritability during CSE is shown as the behavioral response to the distressing stimulus of an inserting endoscopic fiber, and it was measured by the I-CSE scale in this pilot study. This prospective cross-sectional study identified histrionic personality traits, anxiety, and heavy alcohol use to be associative factors for irritability during CSE. Up to our knowledge, this is the first report about the psychological trait associated with irritability during CSE.

According to the psychobiological model of personality, which is known as integrated theory of personality in the contemporary psychiatry, personality consists of temperament and character and could be approached in terms of memory and learning system [24]. Memory can be divided into procedural, semantic, and episodic memory. Procedural memory involves presemantic perceptual processing of information from the physical senses that can operate independently of abstract conceptual and/or volitional processes. Semantic and episodic memories are concerned with factual knowledge and personal contextual awareness of events [25, 26]. Temperament is more relevant with procedural memory and character is more with semantic and episodic memory. Taken into consideration of the state of conscious sedation, responses during CSE are thought to be controlled by procedural memory. In the present study, the difference of responses could be attributed to the difference of temperament between the irritable and stable group.

Patients with histrionic personality have disposition of high novelty seeking temperament [27]. High novelty seeking is characterized by impulsivity, pain-proneness, and vacillating in response to distressing stimuli [27, 28]. Therefore, participants who reported higher histrionic personality traits scores in this study would be expected to have high novelty seeking temperament and to be more impulsive and pain-prone, easily resulting in more irritability in response to endoscope insertion. In line with this, other cluster B personality traits which have high novelty seeking temperament like antisocial/narcissistic/borderline personality in DSM-IV-TR could show excessive irritability during CSE. In the DSM-5 which was released in May 2013, personality disorder is still classified into 10 categories just like DSM-IV-TR. However, section III in the DSM-5 also has an alternative model including the level of personality functioning and five broad domain of personality trait. DSM-5 has a hybrid dimensional/categorical model for personality disorder [29]. It means that more dimensional approach to the personality disorder will emerge apparently in the form like section III of DSM-5. Three-cluster system in the DSM-IV personality disorder may also be viewed as dimensional approach [30]. Consequently, future large-scaled researches would be desirable to involve cluster B trait or disorder including antisocial, borderline, and narcissistic personality rather than a specific personality subtype and consider also the five dimensional domains of personality in the section III of DSM-5.

Anxiety of SCL-90-R which reflected the current psychological status showed significantly higher in the irritable group. In anxious state, amygdala is more activated and brain cortical structures like prefrontal cortex and anterior cingulated cortex are hypoactivated [31–34]. In other words, patients with higher anxiety would be in hyperaroused state of brain. Although all participants reached adequate RASS score for CSE in this study, the irritable group with higher anxiety score was supposed to be more aroused state from the perspectives of neuroscience. Taken into consideration that dose of midazolam administered at the time of insertion was not significantly different between the irritable and stable group (3.31 ± 0.69 versus 3.16 ± 0.73, *P* = 0.434, Table 4), the more anxious participants of irritable group might be more easily awakened and irritable in response to endoscopic stimuli.

Heavy alcohol drinking was the most powerful factor (S.E = 0.632, OR = 4.731, 95% C.I. 1.370–16.337, Table 7).Alcohol enhances the inhibitory neurotransmission at benzodiazepine receptor-sensitive gamma aminobutyric acid (GABA) synapses similar to benzodiazepines; alcohol and benzodiazepine show cross tolerance [35]. If a heavy alcohol drinker receives the same dosage of benzodiazepines as a nonalcohol drinker, the former would likely be less sedated than the latter. Although more dosage of midazolam was needed in the heavy alcohol group for the adequate CSE in this study, the heavy alcohol group was less sedated in terms of RASS than nonalcohol group (Table 8). The result that heavy alcohol drinking was the most powerful associative factor for irritability during CSE is well consistent with a guideline of the American Society for Gastrointestinal Endoscopy for sedation and anesthesia during gastrointestinal endoscopy published in 2008. The guideline recommended that patients such as long-term users of narcotics, benzodiazepines, alcohol, or neuropsychiatric medications require deeper sedation or general anesthesia [36].

This finding that psychological and behavioral factors like personality and alcohol use behavior would be associated with the irritability during CSE may indicate admittance of psychological assessment to daily endoscopic procedures. Taken into consideration of the biopsychosocial model of medical illness [37], it is not surprising that any medical procedure should have the psychosocial component as well as biological one. However, the psychosocial factors have not been integrated with the area of gastrointestinal endoscopic procedures. The present authors hope that this preliminary study would contribute to admit psychosocial element in the practice and facilitate more researches about this topic.

This study has a few limitations. First of all, this is small sample-sized, case-control study. Large-scaled researches are needed to clarify the impact of psychological variables like cluster B personality trait with various covariant on the irritability during CSE in the future. However, it is expected for our pilot study to justify future investigations concerning with this topic. Second, although the I-CSE scale in this study was developed and conducted by a special gastroenterologist who had keen experiences over for a decade in the field of endoscopic procedures, it is recommended in the future research to assess the validity and reliability for I-CSE. Finally, the regional cultural character could have influenced our study; the proportion of irritable patients may be higher in the region where this study was conducted than in others. Moreover, it was not a consecutive case study, so there may be a selection bias for epidemiological purposes. The epidemiological data should be reevaluated in other provinces.

In summary, this study provides the initial assessment of several associative factors for irritability during CSE. Participants with histrionic personality traits, anxiety, or heavy alcohol use may need more careful management during CSE, including considerable preparation, education, and adjustment of midazolam dosages. Moreover, deeper sedation induced by a more potent agent with monitoring using a bispectral index would be helpful for these patients.

## Figures and Tables

**Table 1:** Richmond Agitation-Sedation Scale [21].

Grade	Description	
+4	**Combative**	Combative, violent, immediate danger to staff
+3	**Very agitated**	Pulling or removing tube(s) or catheter(s); aggressive
+2	**Agitated**	Frequent nonpurposeful movement, fights ventilator
+1	**Restless**	Anxious, apprehensive but movements are not aggressive or vigorous
0	**Alert and calm**	
−1	**Drowsy**	Not fully alert, but has sustained awakening to voice (eye opening and contact >10 sec)
−2	**Light sedation**	Briefly awakens to voice (eye opening and contact <10 sec)
−3	**Moderate sedation**	Movement or eye opening to voice (but no eye contact)
−4	**Deep sedation**	No response to voice, but movement or eye opening to physical stimulation
−5	**Unarousable**	No response to voice or physical stimulation

**Table 2:** Irritability during Conscious Sedation Endoscopy Scale (I-CSE).

Grade		Description
0	**No motor response**	
I	**Minimal movement**	Patient turns or moves the head, hands, or feet slightly; no assistant is needed to restrain the patient.
II	**Moderate movement**	Patient shows definite resistance such as turning head and moving arms and calves; one assistant is needed to retrain the patient.
III	**Severe movement**	Patient moves his/her whole boy including head, shoulders, thighs, and trunk; at least two assistants are needed to restrain the patient.
IV	**Fighting against procedure**	Patient is so violent that he/she tries to remove the endoscope with his/her hands. It is impossible to perform procedure.

**Table 3:** Baseline characteristics of irritable and stable groups.

	Irritable group	Stable group	*P*
	*n* = 32	*n* = 32	
Age, mean ± SD, year	56.6 ± 10.8	57.6 ± 12.5	0.702
Male, *n* (%)	17 (53.1)	19 (59.4)	0.614
Body mass index, Kg/m^2^	23.0 ± 8.7	25.0 ± 3.5	0.083
Laboratory findings			
Hemoglobin, g/dL	13.1 ± 1.8	13.2 ± 1.7	0.900
AST, mean ± SD, IU/L	22.4 ± 12.8	22.0 ± 8.3	0.178
ALT, mean ± SD, IU/L	25.0 ± 8.4	20.0 ± 6.0	0.008
BUN, mean ± SD, mg/dL	16.9 ± 5.4	16.0 ± 2.2	0.134
Cr, mean ± SD, mg/dL	0.8 ± 0.6	0.8 ± 0.2	0.657
Na, mean ± SD, mEq/L	137.5 ± 3.2	138.2 ± 2.6	0.448
K, mean ± SD, mEq/L	3.7 ± 0.6	3.9 ± 0.3	0.119
Purpose of endoscopy			
Screening examination, *n* (%)	17 (53.1)	15 (46.9)	0.875
Follow-up after endoscopic	13 (40.6)	15 (46.9)	
Submucosal dissection, *n* (%)			
Dyspepsia, *n* (%)	2 (6.3)	2 (6.3)	
Comorbidity			
Diabetes mellitus, *n* (%)	3 (9.4)	6 (18.8)	0.474
Hypertension, *n* (%)	11 (34.4)	6 (18.8)	0.257
Fatty liver, *n* (%)	16 (50.0)	10 (31.3)	0.127
Heavy alcohol user, *n* (%)	21 (65.6)	9 (28.1)	0.003
Cigarette smoker, *n* (%)	6 (18.8)	13 (40.6)	0.055

**Table 4:** Dose of midazolam and Richmond Agitation-Sedation Scale between irritable and stable group at the time of insertion.

	Irritable group	Stable group	*P*
	*n* = 32	*n* = 32	
Dose of midazolam, mean ± SD, mg	3.31 ± 0.69	3.17 ± 0.73	0.434
Richmond Agitation-Sedation Scale			0.592
−1, *n* (%)	6 (18.8)	4 (12.5)	
−2, *n* (%)	8 (25.0)	11 (34.4)	
−3, *n* (%)	15 (46.9)	16 (50.0)	
−4, *n* (%)	3 (9.4)	1 (3.1)	

**Table 5:** Comparisons of psychological variables between irritable and stable group.

	Irritable group	Stable group	*P*
	*n* = 32	*n* = 32	
Histrionic personality trait, mean ± SD	9.5 ± 3.1	6.9 ± 2.9	0.001
Extravert, *n* (%)	17 (53.1)	12 (37.5)	0.209
SCL-90-R, mean ± SD			
Somatization	48.6 ± 9.9	47.2 ± 8.7	0.549
Obsession-compulsion	46.0 ± 10.2	46.1 ± 11.1	0.879
Interpersonal sensitivity	47.1 ± 8.6	46.5 ± 10.2	0.822
Depression	46.5 ± 7.9	46.7 ± 9.2	0.954
Anxiety	52.8 ± 8.6	46.1 ± 9.6	0.004
Hostility	45.1 ± 6.9	45.8 ± 8.1	0.727
Phobic anxiety	48.4 ± 9.8	46.9 ± 8.3	0.513
Paranoid ideation	46.0 ± 8.3	43.7 ± 7.1	0.232
Psychoticism	47.7 ± 7.0	47.4 ± 7.5	0.877

**Table 6:** Pearson's correlation coefficients between psychological variables and the I-CSE score for the total pooled sample, the irritable group, and the stable group.

	Total pooled sample	Irritable group	Stable group
	*n* = 64	*n* = 32	*n* = 32
Histrionic personality trait	0.334^∗∗^	0.187	−0.038
SCL-90-R			
Somatization	0.144	0.101	0.182
Obsession-compulsion	0.144	0.099	0.409^∗^
Interpersonal sensitivity	0.080	−0.157	0.456^∗∗^
Depression	0.169	0.106	0.456^∗∗^
Anxiety	0.262^∗^	−0.111	0.319
Hostility	0.107	0.143	0.262
Phobic anxiety	0.168	0.058	0.356^∗^
Paranoid ideation	0.121	−0.083	0.273
Psychoticism	0.150	0.148	0.259

^∗∗^ *P* < 0.01; ^∗^ *P* < 0.05.

**Table 7:** Logistic regression analysis showing associated factors with irritability during conscious sedative endoscopy.

	*B*	S.E	Wald statistic	*P*	Exp(*B*)	95% CI	
						Lower	Upper
Histrionic personality trait	0.257	0.105	5.975	0.015	1.294	1.052	1.590
Anxiety	0.088	0.034	6.559	0.010	1.092	1.021	1.168
Heavy alcohol use	1.554	0.632	6.041	0.014	4.731	1.370	16.337

*R* ^2^ = 0.323 (Cox & Snell), 0.430 (Nagelkerke), Model *χ* ^2^(3) = 75.0.

**Table 8:** Dose of midazolam and Richmond Agitation-Sedation Scale between heavy alcohol user and nonheavy alcohol user at the time of insertion.

	Heavy alcohol use	Nonheavy alcohol use	*P*
	*n* = 30	*n* = 34	
Dose of midazolam, mean ± SD, mg	3.48 ± 0.64	3.02 ± 0.70	0.009
Richmond Agitation-Sedation Scale			0.043
−1, *n* (%)	2 (6.7)	2 (5.9)	
−2, *n* (%)	7 (23.3)	3 (8.8)	
−3, *n* (%)	12 (40.0)	7 (20.6)	
−4, *n* (%)	9 (30.0)	22 (64.7)	

## Abbreviations

CSE: Conscious sedation endoscopy
DSM: Diagnostic and Statistical Manual of Mental Disorders
SCID-II: Structured Clinical Interview for DSM-IV-TR Axis II Personality Disorder
MBTI: Myers-Briggs Type Indicator
SCL-90-R: Symptom Checklist-90-Revised
I-CSE: Irritability during conscious sedation endoscopy.
